# Differences in Balance Ability and Motor Control between Dancers and Non-Dancers with Varying Foot Positions

**DOI:** 10.3390/jfmk5030054

**Published:** 2020-07-20

**Authors:** Brooke V. Harmon, Andrea N. Reed, Rebecca R. Rogers, Mallory R. Marshall, Joseph A. Pederson, Tyler D. Williams, Christopher G. Ballmann

**Affiliations:** Department of Kinesiology, Samford University, 800 Lakeshore Dr., Birmingham, AL 35229, USA; bharmon@samford.edu (B.V.H.); areed2@samford.edu (A.N.R.); rrogers1@samford.edu (R.R.R.); mmarshal@samford.edu (M.R.M.); jpederso@samford.edu (J.A.P.); twilli11@samford.edu (T.D.W.)

**Keywords:** motor control test, stability, Y balance test, first position

## Abstract

The purpose of this study was to investigate balance and motor control in dancers and non-dancers with different foot positions. Physically active female dancers (*n* = 11) and non-dancers (*n* = 9) randomly completed two balance tests in a single visit: 1) Y-balance test (YBT), and 2) motor control test (MCT). Each test was completed with two different foot positions: 1) first ballet position in which heels were touching and feet were externally rotated to 140 degrees, and 2) sixth ballet position in which heels were spaced 10 cm apart and forward parallel. For the YBT, participants completed three attempts at anterior, posteromedial, and posterolateral reaches, which were averaged and standardized to limb length for a composite score. For the MCT, participants completed a multi-directional target test on a Biosway balance system, and accuracy and time to completion were analyzed. Findings revealed no differences in YBT score (*p* = 0.255), MCT score (*p* = 0.383), or MCT time (*p* = 0.306) between groups in the sixth position. However, dancers displayed better YBT scores (*p* = 0.036), MCT scores (*p* = 0.020), and faster MCT times (*p* = 0.009) in the first position. Results suggest that superior balance and motor control in dancers may be limited to less innate dance-specific foot positions.

## 1. Introduction

Balance, specifically human balance, is the state of the body in equilibrium with forces acting on it, which allows for the ability not to fall [1]. Different domains of balance may be under static conditions in which center of gravity (CoG) is preserved or during dynamic conditions where equilibrium must be maintained while in motion under a base support [2]. Balance ability may determine performance in highly coordinated sports (i.e., gymnastics/dance, alpine skiing, figure skating) but also predict injury risk, especially in lower extremities [3]. The sport of dance is widely accepted as necessitating balance skill, and balance exercises are highly integrated into training regimens of almost all types of dance. While static balance and stability have generally been shown to be superior in dancers compared to non-dancers, less is known about how balance control differs between them, especially in differing foot positions.

Fitness in dance is unique and primarily requires high levels of coordination and stability control. Interestingly, professional dancers have been reported to have comparable cardiorespiratory fitness to healthy sedentary counterparts despite extensive training and years of dance experience [4]. However, dancers have repeatedly been shown to have better abilities in maintaining different aspects of balance compared to non-dancers. Krtyakiarana et al. showed that classical dancers maintain postural stability to a greater degree than non-dancers, and this difference is accentuated under multitasking conditions [5]. Kilroy et al. reported dancers were able to balance for a longer period of time in a single-legged static stance on both dominant and non-dominant lower extremities [6]. Improvements in stability may be due to higher strength in plantar and hallux flexors when challenged in the anterior–posterior direction [7]. Additionally, hip strength and flexibility have been postulated as potential mechanisms of superior balance in dancers [8].

Assessments of various aspects of balance in dancers have been measured in postural (i.e., CoG), static (i.e., balance error scoring system, single leg stance on force platform), and dynamic (i.e., star excursion balance test, YBT, multiaxial stability index) aspects. Since different forms of dance have distinctive demands, using such tests to evaluate training to improve specific aspects of balance are used by dance athletes and practitioners. Dowse et al. showed improved dynamic balance assessed via multiaxial stability index and lower body strength in dancers undergoing a nine-week resistance training program [9]. Importantly, these improvements accompanied better subjective dance technique, spatial skills, and overall dance performance. Furthermore, proprioceptive–neuromuscular training regimens have been shown to improve single legged static and dynamic balance measures [10]. However, balance tests may also be used a tool to assess or predict injury risk in dancers. Filpa et al. reported that star excursion balance scores predicted function turn out angle and have been used as a marker for lower injury risk [11]. Furthermore, injured dancers have been shown to perform poorer on postural stability tests compared to uninjured dancers [12]. Thus, assessment of balance in dancers has value from a performance and health perspective.

Dance as a sport and artform has distinct physical demands highly reliant on the ability to maintain balance in a variety of different conditions. From a practical standpoint, balance ability may be able to be used to detect risk of injury, thus potentially allowing for information on personalized rehabilitation [13]. To date, most investigations have focused on static and postural stability in dancers and non-dancers in neutral foot positions. Casabona et al. reported that dancers have improved static balance in more difficult dance-specific stances, while dancers and non-dancers had similar performance in more natural foot positions [14]. However, dancing requires various foot positions with motor control and dynamic balance. Thus, understanding the multi-faceted aspects of balance adaptations in dance may provide new insight into mechanisms of training and injury risk assessment. The purpose of this study was to investigate dynamic balance ability and motor control in dancers and non-dancers in the first and sixth ballet positions.

## 2. Materials and Methods 

### 2.1. Study Design

**Figure 1:**
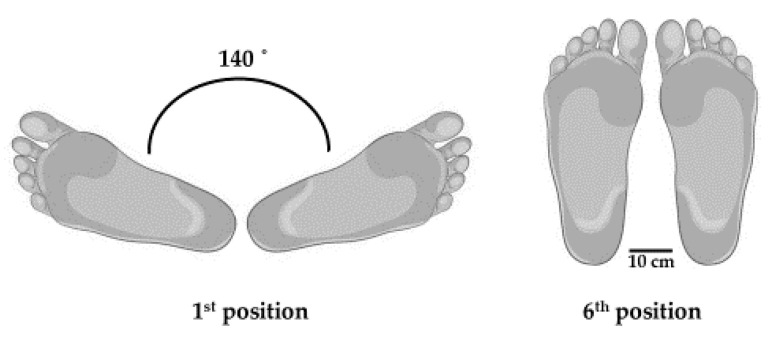
Visual depiction of both 6th and 1st positions used during balance tests. Note: if unipedal, the participant’s single foot serving as the base was still at the same position as shown above as the sole base of support.

In a static groups comparison design, dancers and non-dancers randomly completed two balance tests in a single visit: 1) Y-balance test (YBT), and 2) motor control test (MCT). Each balance test was completed in a modified 1st ballet position (turned out) and 6th ballet position (parallel), which are fundamental ballet foot positions, as previously described by Casabona et al. [14]. For the 1st ballet position, heels were touching, and feet were externally rotated 140 degrees from hallux to hallux (if unipedal, the single foot was rotated 70 degrees). Visual representation of the foot positions can be seen in Figure 1. The YBT was used as a unipedal measure of dynamic balance while the MCT was a bipedal measure of multidirectional motor control. All balance tests were conducted barefoot and by the same two researchers for all measurements.

### 2.2. Participants

Physically active female dancers (*n* = 11) and non-dancers (*n* = 9) were recruited for this study. Descriptive characteristics of participants are presented in Table 1. Physically active was defined as participating in ≥150 min·wk^−1^ of moderate intensity exercise. To be categorized as a dancer, individuals had to have at least 8 years of dance experience in performance or classes (avg dance experience = 15.3 yr ± 2.06) and be actively dancing at the time of the investigation. To determine suitability for exercise, a physical activity readiness questionnaire (PARQ) was used. All participants reported no lower extremity, lumbopelvic, or musculoskeletal injury and no vestibular or balance impairments. All experimental procedures were conducted in accordance with the Declaration of Helsinki and approved by the Samford University Institutional Review Board (EXPD-HP-20-S-3).

### 2.3. Y-balance Test (YBT)

The YBT is a unipedal functional test of dynamic balance. To account for possible differences in limb length, each participant’s limb length (LL) of both left and right lower extremities was measured. To achieve this, participants laid supine and total length in centimeters from the anterior superior iliac spine to the distal edge of the medial malleolus was documented. A Y-balance system (Functional Movement Systems, Lynchburg, VA, USA) consisting of a central platform and three branching tubes marked in ½ cm increments in the anterior, posteromedial, and posterolateral was utilized. On each tube, a sliding indicator block was attached to indicate reach distance during the test. Participants stood in a unipedal stance in corresponding foot positions and performed a maximal reach in each direction while moving the sliding indicator with their toes. The distance reached was recorded to the nearest ½ cm at the proximal edge of the sliding indicator. Participants completed three successful reaches in each direction for both the left and right legs. All reaches were averaged to give a single score in each direction. A successful reach was indicated by an attempt without 1) losing balance during the extension motion and return to the platform, 2) lifting their heel off the contact foot from the platform, 3) losing contact between the reach foot and indicator, and 4) using the indicator as support/putting weight on the indicator [15]. Scores for each leg for both positions were calculated by the following calculation: ((avg anterior + avg posteromedial + avg posterolateral)/(3 × LL) × 100) [15]. Left and right leg scores were then averaged to give a total composite score for 1st and 6th ballet positions.

### 2.4. Biosway Motor Control Test (MCT)

To test motor and balance control, participants completed an MCT protocol on a Biosway portable balance system (Biodex Medical Systems Inc., Shirley, NY, USA) according to the manufacturer’s instructions. In a bipedal stance, participants stood on a balance platform while looking at a screen display with a center point (representing CoG) and eight surrounding targets arranged in a circle. Once the test began, participants shifted and controlled their balance with their feet stationary to hit a randomly blinking target. Once the target was hit, participants had to return their balance back to the center point before another target would be highlighted. This was completed for a single test until each of the 8 targets during the attempt were hit. Participants did this once with the 1st and again with the 6th ballet position. Accuracy, or amount of deviation from the direct path to the target, and time to completion of the test were derived and recorded from the Biosway system.

### 2.5. Data analysis

All data was analyzed using Jamovi software (Version 0.9, Jamovie, Sydney, Australia). Normality of distribution was confirmed using a Shapiro–Wilk test. The comparison of interest was the between groups effect (i.e., dancers versus non-dancers) for each separate foot position. Thus, an independent *t*-test was used to compare measures between groups for each position. Cohen’s d effect sizes (*d*) were calculated as *d* = (M_1_–M_2_)/(SD_pooled_) between groups and interpreted as 0.2—small; 0.5—moderate; and 0.8—large [16,17]. Significance was set at *p* ≤ 0.05 a priori. All data were presented as mean ± standard deviation (SD).

## 3. Results

**Figure 2:**
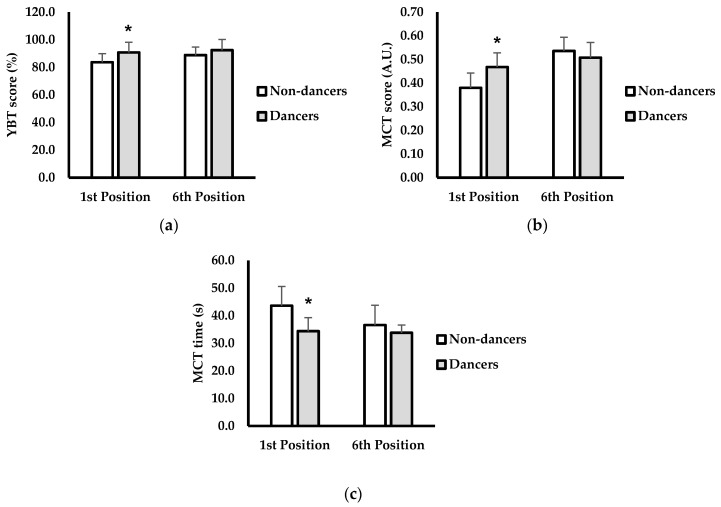
(**a**) Composite Y-balance test (YBT) scores (%) in 1st ballet position and 6th ballet position between non-dancers (white) and dancers (grey); (**b**) Biosway motor control test (MCT) scores (arbitrary units, A.U.) in 1st ballet position and 6th ballet position between non-dancers (white) and dancers (grey); (**c**) MCT completion time (s) in 1st ballet position and 6th ballet position between non-dancers (white) and dancers (grey); data are presented as mean ± SD. * Indicates significantly different from 1st position non-dancer (*p* < 0.05).

Descriptive characteristics are shown in Table 1. Age (years; *p* < 0.001; *d* = 2.27) was significantly higher and body mass (kg; *p* = 0.012; *d* = 1.24) lower in the dance group compared to non-dancers. No differences existed for height (cm; *p* = 0.271; *d* =0.53) or lower limb length (cm; *p* = 0.178; *d* = 0.63). YBT composite scores are shown in Figure 2a. Participants in the dancer group scored significantly higher on the YBT compared to non-dancers when standing in the 1st ballet position (non-dancers = 83.7% ± 5.8, dancers = 90.8% ± 7.7; *p* = 0.036; *d* = 1.2). However, no differences in YBT scores were observed between groups when standing in the sixth ballet position (non-dancers = 88.8% ± 6.1, dancers = 92.4% ± 7.3; *p* = 0.535; *d* = 0.2).

MCT scores and completion times are shown in Figure 2b and Figure 2c, respectively. Dancers scored significantly better than non-dancers on the MCT while standing in the 1st ballet position (non-dancers = 0.38 a.u. ± 0.06, dancers = 0.47 a.u. ± 0.06; *p* = 0.020; *d* = 1.5). MCT scores were not significantly different between dancers and non-dancers when standing in the sixth ballet position (non-dancers = 0.51 a.u. ± 0.06, dancers = 0.54 a.u. ± 0.04; *p* = 0.383; *d* = 0.5). MCT time to completion was significantly faster in the dancer group while standing in the 1st position (non-dancers = 34.3 s ± 4.9, dancers = 43.6 s ± 6.9; *p* = 0.016; *d* = 1.5). However, no significant difference for MCT times existed between dancers and non-dancers in the sixth ballet position (non-dancers = 36.7 s ± 7.1, dancers = 33.7 s ± 2.8; *p* = 0.306; *d* = 0.5).

## 4. Discussion

While previous studies have described superior dynamic balance in dancers [18], information on how varying foot positions influence balance ability is lacking. Findings from this investigation showed no differences in dynamic balance or motor control when standing in the parallel sixth ballet position. However, dancers showed superior dynamic balance performance and motor control while standing in the turned-out first ballet position. While exact mechanisms for adaptations were not currently elucidated, these data add interesting evidence to the body of literature suggesting that dancers’ superior balance and motor control performance may be limited to more dance-specific foot positions.

In the current investigation, dancers and non-dancers performed similarly on the YBT and MCT while standing in the sixth ballet position. This is in stark contrast to previous findings, which have largely shown dancers to have better balance in similar neutral foot positions. Kilroy et al. reported that dancers were able to balance longer and maintain CoG to a greater degree in a unipedal stance compared to non-dancers [6]. However, Kilroy et al. utilized static balance measurements through a force platform, which differed from the unipedal dynamic approach in the current study. Static and dynamic balance require different levels of joint stabilization and abilities to produce muscular force [19]. While joint stiffness and co-contracture of muscles may be important for static balance, weak relationships for stiffness and adaptations often leading to improvements in dynamic balance (i.e., strength, muscle force, etc.) have been reported previously [20]. Since adaptations with dance training may manifest themselves in increased joint stability and stiffness [21], dance training may have a greater impact on unipedal static versus unipedal dynamic balance in neutral foot positions. Supporting this, Ambegaonkar et al. reported that unipedal static stability was higher in dancers versus non-dancers, but balance did not differ in alternating leg dynamic tests and only differed in a portion of directions for dynamic reach tests [22]. In contrast, there have been previous investigations showing superior dynamic balance in young dancers versus non-dancers, necessitating more study for what mechanisms are underlying balance adaptations to static and dynamic conditions differently [18]. Another possible explanation for lack of difference in the sixth position may be due to the familiarity of the position in that most able-bodied individuals will likely stand with feet pointed parallel on a daily basis. Indeed, Casabona et al. reported that more natural neutral foot positions did not alter static balance outcomes in dancers versus non-dancers, while more challenging dance-specific stances caused dancers to perform better [14]. Our findings of no changes in dynamic measures or motor control using natural stances supports this, although there have been other investigations showing better dynamic balance in dancers versus non-dancers with neutral foot positions [18,23]. However, participants in the non-dancer group in the current investigation were still considered physically active. Higher levels of physical activity, both free-living and structured, have been shown to improve balance outcomes [24,25]. Thus, training habits of the non-dancers may have resulted in similar adaptations to dancers, which allowed for similar balance and control in the sixth position. However, differences in regular training habits of dancer and non-dancer groups was not investigated in the current study, leaving the contribution of specific aspects of exercise training unclear. Based on this, future investigations should attempt to delineate specific fitness measures and how they correspond to balance and motor control differently in dancers and non-dancers.

Despite lack of differences in the sixth position, dancers displayed better dynamic balance in the YBT and better motor control in the MCT while standing in the first position. These findings are supported by Casabona et al. who showed that dancers exhibited better static balance while standing in an identical turned out position as the present study but not in neutral positions [14]. Likely, differences between dancers and non-dancers are due to training specificity. Balancing while externally rotated requires high levels of hip and ankle strength and flexibility. Indeed, Gupta et al. showed that dancers have greater hip strength and range of motion compared to non-dancers [8]. Additionally, while individual motor skills may have certain degrees of transferability to others, extensive practice and training may result in more specialized capabilities, which lead to adaptation in the trained skill that may not be generalizable [14,26]. In relation to the current findings, extensive practice in dancers may have led to particularly improved skill in the first position, which is largely a dance-specific skill that did not transfer to superior balance in the sixth position. Important to the current investigation, dancers were more accurate and faster during the MCT compared to non-dancers. This suggests that dancers not only displayed more effective multi-directional balance control but could do so more efficiently. These findings may have practical implications for predicting injury risk. Previous evidence in basketball players reported multi-directional balance performance was predictive of lower extremity injury risk [27]. Furthermore, athletes with higher balance ability before starting the competition season have been shown to be less likely to be injured during the season [28]. While largely speculative based off the current study design, using accuracy and time of motor control tests in various positions in dance could possibly lead to prediction of which foot positions during dance performance may lead to higher rates of injuries in individuals. Supporting this, reduced functional turnout angles in the first ballet position have been shown to be associated with higher numbers and severity of injury in professional dancers [29]. However, injury risk was not measured in the current investigation but could be a practical application for subsequent studies to investigate.

## 5. Conclusions

The current brief report provides novel evidence on balance and motor control between dancers and non-dancers. However, there were several limitations. Although the sample size of the current investigation is in agreement with similar studies [6,14], large samples are warranted in order to maximize generalizability and comparison to other dance populations. Anthropometric features (i.e., body mass, body composition, etc.) were not controlled between groups, and dancers in the present study tended to be lighter than non-dancers. However, sports like dance and gymnastics have been consistently documented to have a tendency of smaller athletes [30]. Total body and lean mass has been shown to influence balance ability; thus, we cannot rule out the possibility of body composition affecting the results [31]. Furthermore, very recent evidence has shown that YBT performance may be related to trunk and lower limb strength [32]. Unfortunately, this specific information was not obtained with the current study design, so present YBT results should be viewed with optimism but also caution. More study is needed to investigate body characteristic differences between dancers and non-dancers and how they may specially alter balance and motor control ability. Additionally, no adaptive mechanisms possibly contributing to changes in balance performance were measured. Given that numerous factors including proprioception, flexibility, and muscular strength may predict balance ability [7,33], more specific study on which factors may contribute to changes in performance with different foot positions is needed. In conclusion, dance participation and experience may not influence balance and motor control in the sixth ballet position but result in better balance outcomes while standing in the first ballet position. These findings provide new information pertaining to functional performance in dancers and may have implications for identifying specific training adaptations and injury risk in varying foot positions.

## Figures and Tables

**Table 1:** Descriptive characteristics of participants. * indicated significantly different from non-dancer (*p* < 0.05). Data are presented as mean ± SD.

Characteristic	Non-Dancer (*n* = 9)	Dancer (*n* = 11)
Age (yr)	19.5 ± 0.8	21.2 ± 0.7 *
Height (cm)	163.2 ± 3.9	165.3 ± 3.9
Body mass (kg)	64.3 ± 8.1	58.1 ± 6.3 *
Lower limb length (cm)	88.2 ± 4.54	85.9 ± 4.8
